# Smartphone Monitoring of Gait and Balance During Irregular Surface Walking and Obstacle Crossing

**DOI:** 10.3389/fspor.2020.560577

**Published:** 2020-11-27

**Authors:** Janeesata Kuntapun, Patima Silsupadol, Teerawat Kamnardsiri, Vipul Lugade

**Affiliations:** ^1^Department of Physical Therapy, Faculty of Associated Medical Sciences, Chiang Mai University, Chiang Mai, Thailand; ^2^College of Arts, Media and Technology, Chiang Mai University, Chiang Mai, Thailand; ^3^Control One LLC, Atlanta, GA, United States

**Keywords:** smartphones, gait, irregular surfaces, obstacle crossing, elderly

## Abstract

As gait adaptation is vital for successful locomotion, the development of field-based tools to quantify gait in challenging real-world environments are crucial. The aims of this study were to assess the reliability and validity of a smartphone-based gait and balance assessment while walking on unobstructed and obstructed terrains using two phone placements. Furthermore, age-related differences in smartphone-derived gait strategies when navigating different walking conditions and environments were evaluated. By providing a method for evaluating gait in the simulated free-living environment, results of this study can elucidate the strategies young and older adults utilize to navigate obstructed and unobstructed walking paths. A total of 24 young and older adults ambulated indoors and outdoors under three conditions: level walking, irregular surface walking, and obstacle crossing. Android smartphones placed on the body and in a bag computed spatiotemporal gait (i.e., velocity, step time, step length, and cadence) and balance (i.e., center of mass (COM) displacement), with motion capture and video used to validate parameters in the laboratory and free-living environments, respectively. Reliability was evaluated using the intraclass correlation coefficient and validity was evaluated using Pearson's correlation and Bland-Altman analysis. A three-way ANOVA was used to assess outcome measures across group, condition, and environment. Results showed that smartphones were reliable and valid for measuring gait across all conditions, phone placements, and environments (ICC_2,1_: 0.606–0.965; Pearson's *r*: 0.72–1.00). Although body and bag placement demonstrated similar results for spatiotemporal parameters, accurate vertical COM displacement could only be obtained from the body placement. Older adults demonstrated a longer step time and lower cadence only during obstacle crossing, when compared to young adults. Furthermore, environmental differences in walking strategy were observed only during irregular surface walking. In particular, participants utilized a faster gait speed and a longer step length in the free-living environment, compared to the laboratory environment. In conclusion, smartphones demonstrate the potential for remote patient monitoring and home health care. Along with being easy-to-use, inexpensive, and portable, smartphones can accurately evaluate gait during both unobstructed and obstructed walking, indoors and outdoors.

## Introduction

Gait performance is an important marker of functional ability, independent living, and survival (Hardy et al., 2007; Lord et al., 2013). In particular, the ability to modify gait to suit different environmental contexts is vital for successful locomotion, especially in complex environments such as irregular surfaces or negotiating obstacles. To examine gait adaptation, most research replicated these challenging walking situations in a low-distraction laboratory setting using sophisticated equipment (i.e., motion capture systems or instrumented walkways) (Chen et al., 1994; Weerdesteyn et al., 2005; Marigold and Patla, 2008). However, these conventional systems cannot collect data from more than a few gait cycles, thus may not reflect the usual gait behavior of people during daily life.

Recently, the use of body-worn inertial measurement units (IMU) has allowed for continuous, remote data collections over extended time periods (Patel et al., 2012). Although IMUs have demonstrated excellent concurrent validity for assessing gait compared to conventional instruments (Hartmann et al., 2009), they have numerous shortcomings. Attachment of an IMU directly onto the body can lead to discomfort, and thus reduce compliance (del Rosario et al., 2015). Importantly, an IMU has a relatively high cost for commercial software packages and requires trained personnel to perform the assessment, operate the system, and interpret the data. Smartphones have thus been proposed as a potential instrument for gait evaluation. With the ubiquity of smartphones, utilizing the embedded sensors and user-friendly interface of a smartphone can allow for a cost-effective, convenient, and automated tool for assessing gait among health care providers, care givers, athletes, or patients.

Current smartphone technology has made remote and prolonged gait assessment possible. Previous studies have shown that a smartphone placed on the body or in a bag is valid and reliable for measuring spatiotemporal gait parameters during straight walking (Silsupadol et al., 2017), turning, and gait speed modulation (Silsupadol et al., 2019). While spatiotemporal gait parameters provide a basic quantification of walking quality, the center of mass (COM) motion has been used as a global measure of balance control during gait. To date, only one study has investigated the reliability and validity of the vertical COM displacement using a smartphone-based accelerometer (Furrer et al., 2015). The results demonstrated good reliability and moderate correlations when comparing the vertical COM displacement derived from a smartphone-based accelerometer against a motion capture system during level walking.

Validation of smartphone-based gait and balance assessment is currently limited to unobstructed walking. Whether the smartphone-derived validity during unobstructed walking is transferable to challenging real-world walking situations, requires further investigation. It is crucial to assess gait and balance performance under conditions that individuals, especially older adults, are likely to encounter in daily life. Aging is accompanied by deterioration in physical, cognitive, and psychological health that are essential for effective propulsion and balance control, consequently putting the elderly at a greater risk for falls. In particular, a large proportion of falls in older people occur outdoors (51–81%) and are initiated by tripping either on irregular walking surfaces (44%) or on a discrete object (37%) (Li et al., 2006; Robinovitch et al., 2013).

Since gait adaptation is a key requirement for successful locomotion and falls among older adults mostly occur outdoors while walking on irregular surfaces and negotiating obstacles, it is essential to have a valid, user-friendly, and cost-effective measurement system that can quantitatively and continuously assess gait in the free-living environment. As phones are generally carried in different locations, the effect of placement requires investigation. Furthermore, no studies have utilized smartphones to investigate age-related gait and balance performance during a variety of challenging conditions. Therefore, the purposes of this study were: (1) to assess the reliability and validity of a smartphone-based accelerometer in determining spatiotemporal gait and COM displacement during level walking, irregular surface walking (continuous perturbation), and obstacle crossing (discrete perturbation) in both laboratory and free-living environments; (2) to examine inter-placement validity of two identical Android smartphones placed on the body and in a bag; and (3) to evaluate differences in walking strategy among young and older adults across walking condition and environment. We hypothesized that a smartphone-based evaluation of spatiotemporal gait parameters and COM displacement would be reliable and valid across walking conditions and environments, when placed either on the body or in a bag. We further hypothesized that a smartphone-based assessment would elucidate age-related differences in gait performance across environment when negotiating various walking surfaces and obstacles.

## Materials and Methods

### Participants

Young (18–35 years old) and older (65 years or older) adults were recruited into the study through leaflets and announcement through community leaders and primary health care providers. All participants were able to walk at least 10-m without the assistance of another person or walking aid. Exclusion criteria included unstable medical conditions, having visual impairment uncorrectable with lenses, and having lower limb amputation or arthroplasty. This study was approved by the university's research ethics committee (number AMSEC-61EX-043). All participants provided written informed consent prior to enrollment.

### Experimental Design

**Figure 1:**
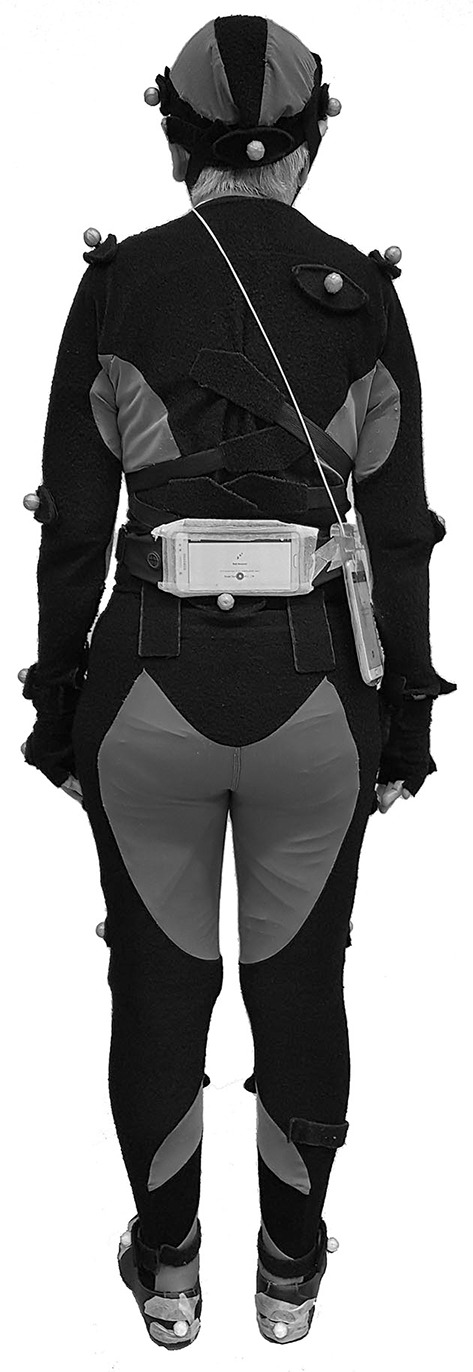
Placement of the two smartphones in a shoulder bag and on the lumbar spine during all walking trials. During laboratory walking trials, reflective markers were placed on bony landmarks of the body while the participant wore a body suit.

Following informed consent, participants' demographic and anthropometric information such as age, gender, and leg length were recorded. During laboratory trials, a total of 28 retro-reflective markers were positioned on bony landmarks of the body, using a modified Helen Hayes marker set (Hahn and Chou, 2004). Participants' gait was evaluated using a nine-camera motion analysis system (Motion Analysis Corp., Santa Rosa, CA), which collected three-dimensional marker trajectories at 120 Hz. In the free-living environment, a video camera recorded participants' gait at 30 Hz. During all walking trials, participants carried two Android smartphones (Samsung J7+ Android 7.1.1; 152.4 × 74.7 × 7.9 mm). One smartphone was attached horizontally at the level of the third lumbar vertebrae using a secure belt pouch (Figure 1). The second smartphone was placed vertically inside a shoulder bag (13 × 20 cm) and rested on the right hip.

**Figure 2:**
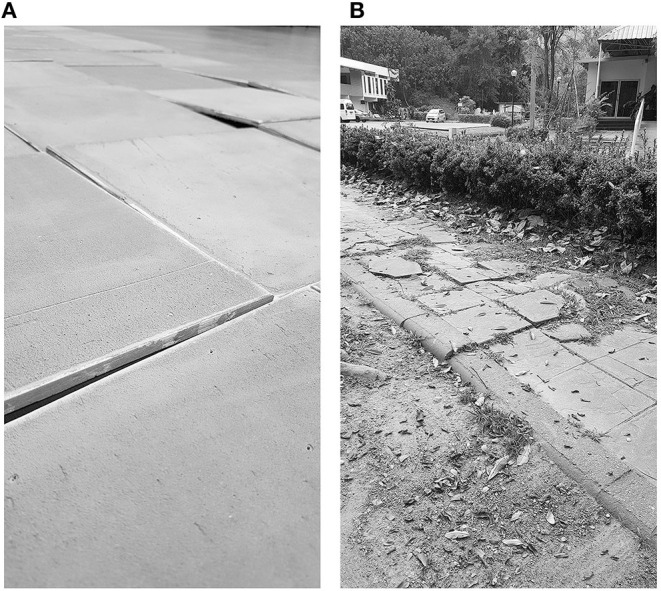
Configuration of the uneven surface while walking in the laboratory **(A)** and in the free-living environment **(B)**.

Participants were asked to walk 10 m at their self-selected comfortable speed under three conditions indoors and outdoors: (1) level walking; (2) irregular surface walking; and (3) obstacle crossing. The controlled laboratory environment was quiet and clutter-free, whereas the simulated free-living environment included a level pedestrian walkway connected to a parking lot, coffee shop, and classrooms. For the irregular surface task performed in the laboratory, a walkway was constructed from 16 irregular terrains (0.6 × 1.2 m) to create a 9.6 × 1.2 m grid. Each terrain consisted of eight plywood pieces (width × height × thickness of 30 × 30 × 0.9 cm) elevated from a level surface using dowels of 0.7–1.6 cm to produce anterior-posterior (AP) or medial-lateral (ML) disturbances (Figure 2A). For the irregular surface condition in the free-living environment, an uneven 10-m portion of a sidewalk located near a parking lot and street was utilized (Figure 2B). For both environments, during the crossing over an obstacle trial, a light-weight plastic obstacle set at a height of 20 cm was placed in the middle of the walkway, to resemble a curb height found in real-life situations.

With three trials performed for each of the three walking conditions across two environments, a total of 18 trials were performed by each participant, with all trials completed during a single visit. The order of all conditions was randomized, with the order of environmental setting counterbalanced. Mean values for each walking condition were used for further analysis.

### Data Acquisition and Analysis

A custom-built Android application, Gait Analyzer (Lugade, 2018), was utilized to collect the smartphone's tri-axial accelerometer (STM LSM6DSL, 39.2 m/s^2^ range; 1.20 × 10^−3^ m/s^2^ resolution) data at 50 Hz. All acceleration data were downloaded following completion of the data collection and analyzed offline using custom written programs in MATLAB (Mathworks Inc., Natick, MA).

Data across all three axes were filtered using a 4th order low-pass Butterworth filter with a 20 Hz cutoff frequency. AP accelerations were further filtered using a Butterworth 4th order low-pass filter with a cutoff frequency of 2 Hz. Positive peaks in the filtered AP direction were used to identify heel strikes. Gait measures were calculated from the acceleration data and heel strike timing events, with this methodology previously shown to be valid and reliable (Zijlstra, 2004; Silsupadol et al., 2017). Step time was defined as the time difference between heel strikes. Step length was calculated as the displacement along the vertical axis across each step cycle and a participant's leg length using the relationship:

(1) $$\begin{array}{r} steplength=2*\sqrt{2*h*l-h^{2}} \end{array}$$

where *h* is the vertical displacement of the center of mass across each step cycle and *l* is the leg length (Zijlstra and Hof, 2003). Step velocity was computed as the quotient of the step length and step time, with gait velocity being the average step velocity across all steps. Cadence was calculated as the number of steps taken over the total trial time.

Smartphone-derived COM displacement was calculated by first removing gravity from the raw acceleration signal by applying a high-pass 3rd order 0.25 Hz elliptical filter with a 0.01 dB passband and 100 dB stopband (Lugade et al., 2014). The body-motion component of acceleration was then numerically double integrated across each step cycle to obtain the velocity and position (Furrer et al., 2015). In order to remove integration drift, a first order recursive high-pass Butterworth filter was applied to both the velocity and position data, with a 0.2 Hz cutoff frequency applied to the AP and ML directions, and 0.5 Hz used for the superior-inferior (SI) direction (Floor-Westerdijk et al., 2012). The COM range of motion was computed as the average of each minimum to maximum displacement in the AP, ML, and SI directions across all step cycles.

Marker data from the three-dimensional motion capture system were downloaded and analyzed using custom written MATLAB programs, with position data initially filtered using a 4th order low-pass Butterworth filter with an 8 Hz cutoff frequency. Heel strikes were detected using the vertical velocity of the midfoot (O'Connor et al., 2007). Step length and step time were calculated using the position and time change of the heel marker between heel strikes. Gait velocity and cadence were computed identically to the methods used for the smartphone analysis. COM was computed as the weighted sum of a 13-segment model, which included segmental COM for bilateral feet, bilateral shanks, bilateral thighs, pelvis, trunk, bilateral upper arms, bilateral lower arms and hands, and the head (Lugade et al., 2011).

Synchronization between motion capture and smartphone was performed by counting the number of steps taken from the starting point till the participant entered the capture volume of the motion capture system. Only steps collected within the capture volume were used for validation purposes. For obstacle crossing trials, only the two crossing steps were used for analysis.

A video editing program (Avidemux 2.7) was used to identify heel strikes of each step from video, and subsequently calculate step time and cadence during free-living trials. Video-based gait assessment revealed excellent intra-rater reliability (ICC_3, 1_ = 0.928) from a single investigator who re-assessed step times of a single participant 1 week after the initial evaluation. Gait velocity was calculated based on the total distance traveled and time elapsed per trial. Synchronization between the video camera and the smartphone was performed using the onset of movement detected by both systems. For level and irregular surface walking, all steps except for the first two gait initiation steps and the final two gait termination steps were included for analysis. Validity and reliability of crossing speed during the obstacle crossing condition could not be evaluated using video-derived data.

### Statistical Analysis

For intra-session reliability, all derived parameters from the two smartphones, motion capture system, and video camera were assessed across trials using the intraclass correlation coefficient (ICC_2,1_). For ICC values, Cicchetti's guidelines was used to interpret results, with values <0.40 representing poor reliability, 0.40–0.59 fair reliability, 0.60–0.74 good reliability, and values >0.75 having excellent reliability (Cicchetti, 1994). For concurrent validity, average values of the data obtained by the different measurement devices were examined using Pearson correlation coefficients (r), with motion capture and video observation considered the gold standard measures in the laboratory and free-living environments, respectively. Correlation *r*-values were interpreted as <0.30 being negligible, 0.30–0.50 low, 0.50–0.70 moderate, 0.70–0.90 high, and 0.90–1.00 very high (Mukaka, 2012). Bland-Altman analysis was used to evaluate the bias and 95% limits of agreement between the smartphone-based evaluations and motion capture or video.

For participant demographics, *t*-tests were used to evaluate differences in age, weight, and height between the young and older groups. A three-way mixed-effects ANOVA was used to investigate differences in gait and balance measures with walking condition and environment setting as within-subject factors, and group as the between-subjects factor. If a three-way interaction was non-significant, all possible two-way interactions were investigated. Main effects were considered only if no two-way interactions were found. If there were any interaction effects, the differences were then estimated at each level. Following statistical guidelines (Prescott, 2019), significance was set at an alpha level of *p* < 0.05 with unadjusted *p*-values reported. Outliers which were not the result of technical error were included in the analysis. All data were analyzed using SPSS 17.0 (IBM Inc., Armonk, NY).

## Results

Twelve healthy young [three males; age 23.4 (2.2) years; weight 58.3 (9.9) kg; height 1.63 (0.07) m] and twelve healthy older adults [three males; 75.6 (5.6) years; 58.0 (6.6) kg; 1.60 (0.09) m] were included in the study. While an age difference was found (*p* < 0.001), there were no height (*p* = 0.40) or weight (*p* = 0.92) differences between groups.

### Reliability

Spatiotemporal gait parameters obtained from motion capture, video, and the two smartphones demonstrated excellent reliability during level and irregular surface walking in both laboratory (ICC_2,1_ ranging from 0.944 to 0.989, 0.908 to 0.965, and 0.882 to 0.953 for motion, smartphone on the body, and smartphone in the bag, respectively; Table 1) and free-living environments (ICC_2,1_ ranging from 0.951 to 0.969, 0.896 to 0.952, and 0.774 to 0.882 for video, smartphone on the body, and smartphone in the bag, respectively; Table 2). While motion capture and video also demonstrated excellent reliability for spatiotemporal gait parameters during obstacle crossing trials, the smartphones showed good to excellent reliability for this condition (ICC_2,1_ ranging from 0.606 to 0.804 and 0.635 to 0.764 for the body and bag placements, respectively). Among the COM displacement parameters, poor to excellent reliability was found for motion capture (ICC_2,1_ ranging from 0.344 to 0.891), fair to excellent reliability for the smartphone placed on the body (ICC_2,1_ ranging from 0.543 to 0.921), and poor to good reliability for the smartphone placed in the shoulder bag (ICC_2,1_ ranging from 0.265 to 0.711).

**Table 1:** Validity and reliability of smartphone-based measures in the laboratory.

**Condition**	**Motion**		**Smartphone-body**				**Smartphone-bag**			
	**Mean (SD)**	**ICC (2,1)**	**Mean (SD)**	**ICC (2,1)**	**r**	**Bias (LOA)**	**Mean (SD)**	**ICC (2,1)**	**r**	**Bias (LOA)**
**Gait velocity (m/s)**										
Level	1.18 (0.21)	0.987	1.27 (0.24)	0.965	0.92	0.09 (−0.10–0.28)	1.24 (0.24)	0.948	0.92	0.06 (−0.12–0.24)
Irregular	1.12 (0.24)	0.972	1.21 (0.26)	0.938	0.95	0.09 (−0.06–0.25)	1.20 (0.25)	0.940	0.95	0.08 (−0.07–0.24)
Obstacle	0.85 (0.20)	0.932	1.03 (0.23)	0.770	0.90	0.18 (−0.02–0.38)	1.04 (0.23)	0.635	0.86	0.19 (−0.04–0.42)
**Step time (ms)**										
Level	527 (47)	0.965	528 (47)	0.964	1.00	1.2 (−5.6–8.0)	531 (48)	0.947	1.00	4.4 (−4.2–13.0)
Irregular	553 (60)	0.944	554 (60)	0.946	1.00	1.0 (−5.9–7.9)	552 (55)	0.882	0.99	−0.6 (−18.5–17.2)
Obstacle	749 (133)	0.901	705 (103)	0.802	0.94	−44 (−143–54)	692 (107)	0.727	0.87	−56 (−185–72)
**Step length (cm)**										
Level	61.3 (8.2)	0.989	66.2 (10.1)	0.954	0.86	4.9 (−5.1–15.0)	65.0 (9.7)	0.953	0.87	3.8 (−5.7–13.2)
Irregular	60.6 (9.4)	0.971	66.0 (10.5)	0.908	0.91	5.4 (−3.1–13.9)	65.5 (10.6)	0.920	0.91	4.8 (−3.6–13.3)
Obstacle	60.4 (9.2)	0.875	70.8 (10.9)	0.606	0.81	10.5 (−2.1–23.0)	70.5 (12.0)	0.654	0.72	10.2 (−6.2–26.5)
**Cadence (steps/min)**										
Level	115 (10)	0.967	114 (10)	0.965	1.00	−0.2 (−1.7–1.2)	114 (10)	0.951	1.00	−0.9 (−2.8–1.0)
Irregular	110 (11)	0.949	110 (11)	0.951	1.00	−0.2 (−1.8–1.4)	110 (10)	0.903	1.00	−0.0 (−2.9–2.9)
Obstacle	82 (12)	0.930	87 (11)	0.761	0.92	4.6 (−4.7–14.0)	89 (13)	0.681	0.88	6.6 (−5.6–18.8)
**COM AP (cm)**										
Level	5.7 (2.4)	0.344	6.0 (1.8)	0.921	−0.30	0.2 (−6.5–7.0)	5.5 (1.8)	0.484	−0.20	−0.3 (−6.8–6.3)
Irregular	6.5 (2.9)	0.461	6.0 (1.9)	0.907	−0.42	−0.5 (−8.5–7.5)	5.8 (1.7)	0.265	0.08	−0.6 (−6.9–5.6)
Obstacle	7.0 (1.9)	0.478	6.4 (2.0)	0.610	0.24	−0.7 (−5.4–4.0)	6.4 (1.8)	0.360	0.29	−0.6 (−4.9–3.7)
**COM ML (cm)**										
Level	6.3 (1.8)	0.488	2.9 (1.2)	0.752	0.14	−3.4 (−7.3–4.4)	6.4 (2.4)	0.556	−0.30	0.1 (−6.6–6.8)
Irregular	7.2 (2.3)	0.556	3.2 (1.0)	0.638	0.12	−4.0 (−8.7–0.7)	6.6 (3.0)	0.711	−0.37	−0.6 (−9.2–8.0)
Obstacle	5.3 (2.1)	0.714	5.0 (2.6)	0.715	0.70	−0.4 (−4.0–3.3)	7.0 (2.9)	0.692	−0.20	1.6 (−6.1–9.3)
**COM SI (cm)**										
Level	4.1 (0.8)	0.891	3.0 (0.5)	0.767	0.63	−1.1 (−2.3–0.1)	3.5 (1.0)	0.573	0.19	−0.6 (−2.8–1.6)
Irregular	4.5 (0.8)	0.805	3.2 (0.7)	0.703	0.75	−1.3 (−2.4–0.2)	3.9 (1.7)	0.676	0.42	−0.7 (−3.6–2.3)
Obstacle	7.9 (1.2)	0.722	4.9 (1.0)	0.543	0.49	−3.1 (−5.4– −0.8)	3.4 (1.0)	0.313	0.19	−4.6 (−7.3– −1.8)

*COM, Center of mass; AP, Anterior-posterior; ML, Medial-lateral; SI, Superior-inferior; LOA, Limits of agreement*.

**Table 1a:** Validity and reliability of smartphone-based measures among young adults in the laboratory.

**Condition**	**Motion**		**Smartphone-body**				**Smartphone-bag**			
	**Mean (SD)**	**ICC (2,1)**	**Mean (SD)**	**ICC (2,1)**	**r**	**Bias (LOA)**	**Mean (SD)**	**ICC (2,1)**	**r**	**Bias (LOA)**
**Gait velocity (m/s)**										
Level	1.26 (0.16)	0.980	1.31 (0.20)	0.953	0.89	0.05 (−0.14–0.24)	1.29 (0.21)	0.910	0.90	0.03 (−0.16–0.21)
Irregular	1.24 (0.17)	0.964	1.31 (0.20)	0.924	0.89	0.07 (−0.10–0.25)	1.31 (0.21)	0.960	0.89	0.07 (−0.12–0.26)
Obstacle	0.98 (0.11)	0.823	1.12 (0.19)	0.821	0.88	0.14 (−0.07–0.36)	1.14 (0.16)	0.539	0.76	0.16 (−0.05–0.37)
**Step time (ms)**										
Level	522 (36)	0.970	523 (37)	0.969	0.99	1.7 (−6.2–9.6)	525 (37)	0.918	1.00	3.4 (−4.1–10.9)
Irregular	533 (38)	0.940	534 (37)	0.945	0.99	1.1 (−8.0–1.0)	533 (36)	0.934	0.99	0.8 (−1.0–11.5)
Obstacle	681 (60)	0.891	654 (57)	0.579	0.82	−27.6(−96.9–41.7)	634 (56)	0.478	0.76	−47.2(−126–31.3)
**Step length (cm)**										
Level	65.3 (6.7)	0.984	68.3 (10.1)	0.946	0.86	3.0 (−7.6–13.6)	67.3 (9.8)	0.935	0.88	1.9 (−7.9–11.7)
Irregular	65.7 (7.1)	0.973	69.8 (9.5)	0.893	0.87	4.2 (−5.4–13.8)	69.5 (9.9)	0.962	0.87	3.9 (−6.0–13.8)
Obstacle	65.4 (8.0)	0.818	72.8 (11.9)	0.852	0.84	7.4 (−5.9–20.7)	72.3 (12.6)	0.784	0.84	6.9 (−7.5–21.2)
**Cadence (steps/min)**										
Level	115 (8.1)	0.973	115 (8.2)	0.970	0.99	−0.4 (−2.0–1.3)	115 (8.2)	0.928	1.00	−0.7 (−2.2–0.8)
Irregular	113 (8.2)	0.934	113 (7.8)	0.945	0.99	−0.3 (−2.5–2.0)	113 (7.7)	0.935	0.99	−0.2 (−2.8–2.3)
Obstacle	89 (8.3)	0.915	93 (8.2)	0.544	0.80	4.0 (−6.2–14.2)	96 (8.7)	0.495	0.79	6.9 (−3.9–17.7)
**COM AP (cm)**										
Level	4.8 (1.2)	0.002	6.2 (1.8)	0.942	0.15	1.5 (−2.5–5.5)	5.9 (2.1)	0.623	−0.09	1.2 (−3.8–6.1)
Irregular	4.8 (0.8)	−0.148	6.5 (0.8)	0.925	0.20	1.7 (−1.8–5.2)	5.3 (1.1)	0.202	0.33	0.5 (−1.8–2.8)
Obstacle	6.1 (1.1)	0.278	6.1 (2.1)	0.690	0.57	−0.0 (−3.4–3.3)	5.9 (1.6)	0.351	0.73	−0.2 (−2.4–1.9)
**COM ML (cm)**										
Level	5.5 (0.6)	−0.022	2.5 (0.7)	0.632	−0.05	−3.0 (−4.8– −1.2)	6.7 (2.4)	0.509	−0.40	1.3 (−4.0–6.5)
Irregular	5.8 (1.3)	0.576	2.8 (0.6)	0.521	−0.24	−3.0 (−6.2–0.1)	6.6 (1.4)	0.569	−0.43	0.8 (−3.8–5.4)
Obstacle	4.3 (0.9)	0.457	3.7 (1.4)	0.489	0.36	−0.6 (−3.4–2.1)	6.8 (2.4)	0.594	0.18	−2.4 (−2.2–7.1)
**COM SI (cm)**										
Level	4.3 (0.8)	0.904	3.1 (0.4)	0.626	0.40	−1.2 (−2.7–0.3)	3.7 (0.8)	0.273	0.06	−0.6 (−2.7–1.6)
Irregular	4.8 (0.7)	0.775	3.4 (0.6)	0.437	0.60	−1.4 (−2.5– −0.3)	3.6 (0.7)	0.379	−0.23	−1.2 (−3.4–0.9)
Obstacle	7.9 (1.3)	0.845	4.7 (0.8)	0.474	0.62	−3.2 (−5.3– −1.2)	3.7 (1.1)	0.468	0.35	−4.2 (−6.9– −1.4)

*COM, Center of mass; AP, Anterior-posterior; ML, Medial-lateral; SI, Superior-inferior; LOA, Limits of agreement*.

**Table 1b:** Validity and reliability of smartphone-based measures among older adults in the laboratory.

**Condition**	**Motion**		**Smartphone-Body**				**Smartphone- Bag**			
	**Mean (SD)**	**ICC (2,1)**	**Mean (SD)**	**ICC (2,1)**	**r**	**Bias (LOA)**	**Mean (SD)**	**ICC (2,1)**	**r**	**Bias (LOA)**
**Gait velocity (m/s)**										
Level	1.10 (0.23)	0.988	1.22 (0.28)	0.972	0.96	0.13 (−0.03–0.29)	1.19 (0.26)	0.973	0.96	0.09 (−0.06–0.25)
Irregular	0.99 (0.24)	0.963	1.11 (0.27)	0.932	0.98	0.11 (−0.01–0.24)	1.09 (0.25)	0.910	0.97	0.10 (−0.02–0.22)
Obstacle	0.73 (0.20)	0.915	0.94 (0.24)	0.696	0.94	0.21 (0.04–0.38)	0.94 (0.26)	0.614	0.88	0.22 (−0.03–0.46)
**Step time (ms)**										
Level	532 (57)	0.965	533 (57)	0.964	1.00	0.6 (−5.0–6.3)	538 (57)	0.962	1.00	5.4 (−4.1–15.0)
Irregular	573 (73)	0.939	574 (73)	0.941	1.00	0.9 (−3.2–5.0)	571 (65)	0.854	0.99	−2.1 (−25.3–21.1)
Obstacle	816 (153)	0.871	755 (116)	0.806	0.94	−60.6 (−175–54.2)	751 (115)	0.694	0.84	−65.3 (−23.2–101)
**Step length (cm)**										
Level	57.2 (7.7)	0.988	64.1 (10.1)	0.962	0.92	6.9 (−1.3–15.0)	62.8 (9.5)	0.971	0.91	5.6 (−2.3–13.5)
Irregular	55.6 (8.9)	0.954	62.2 (10.5)	0.904	0.95	6.6 (−0.3–13.6)	61.4 (10.1)	0.863	0.95	5.8 (−0.7–12.3)
Obstacle	55.3 (7.5)	0.847	68.8 (10.0)	0.382	0.91	13.5 (4.9–22.2)	68.8 (11.7)	0.541	0.71	13.5 (−2.7–29.7)
**Cadence (steps/min)**										
Level	114 (11.8)	0.966	114 (11.8)	0.965	1.00	−0.1 (−1.4–1.2)	113 (11.7)	0.965	1.00	−1.1 (−3.3–1.1)
Irregular	106 (12.2)	0.950	106 (12.1)	0.948	1.00	−0.2 (−0.9–0.5)	106 (11.1)	0.878	0.99	0.2 (−3.1–3.5)
Obstacle	76 (12.8)	0.905	81 (10.9)	0.805	0.94	5.3 (−3.3–14.0)	82 (12.6)	0.648	0.84	6.3 (−7.6–20.2)
**COM AP (cm)**										
Level	6.7 (3.0)	0.369	5.7 (1.8)	0.902	−0.46	−1.0 (−9.1–7.1)	5.0 (1.5)	0.255	−0.15	−1.7 (−0.9–5.3)
Irregular	8.1 (3.2)	0.382	5.5 (2.1)	0.891	−0.51	−2.6 (−11.7–6.4)	6.4 (2.0)	0.252	−0.23	−1.8 (−9.9–6.4)
Obstacle	7.9 (2.1)	0.425	6.6 (2.0)	0.544	0.02	−1.3 (−6.9–4.4)	6.9 (1.9)	0.330	−0.04	−1.0 (−6.7–4.7)
**COM ML (cm)**										
Level	7.1 (2.1)	0.487	3.3 (1.4)	0.757	−0.05	−3.8 (−9.0–1.3)	6.0 (2.5)	0.617	−0.28	−1.1 (−8.5–6.2)
Irregular	8.5 (2.3)	0.387	3.6 (1.2)	0.610	−0.19	−4.9 (−10.3–0.5)	6.6 (4.1)	0.745	−0.49	−2.0 (−12.8–8.9)
Obstacle	6.3 (2.5)	0.688	6.3 (2.9)	0.701	0.65	−0.1 (−4.5–4.4)	7.1 (3.5)	0.757	−0.38	0.8 (−9.0–10.6)
**COM SI (cm)**										
Level	3.9 (0.7)	0.870	2.9 (0.6)	0.860	0.79	−1.0 (−1.9– −0.2)	3.3 (1.1)	0.883	0.20	−0.6 (−2.9–1.7)
Irregular	4.2 (0.9)	0.792	3.1 (0.8)	0.892	0.81	−1.1 (−2.1– −0.1)	4.1 (2.3)	0.719	0.77	−0.1 (−3.4–3.3)
Obstacle	8.0 (1.2)	0.614	5.0 (1.2)	0.582	0.42	−2.9 (−5.4– −0.4)	3.0 (0.7)	0.016	0.01	−5.0 (−7.6– −2.3)

*COM, Center of mass; AP, Anterior-posterior; ML, Medial-lateral; SI, Superior-inferior; LOA, Limits of agreement*.

**Table 2:** Validity and reliability of smartphone-based measures in the free-living environment.

**Condition**	**Video**		**Smartphone-body**				**Smartphone-bag**			
	**Mean (SD)**	**ICC (2,1)**	**Mean (SD)**	**ICC (2,1)**	**r**	**Bias (LOA)**	**Mean (SD)**	**ICC (2,1)**	**r**	**Bias (LOA)**
**Gait velocity (m/s)**										
Level	1.20 (0.25)	0.969	1.16 (0.22)	0.896	0.86	−0.04 (−0.27–0.21)	1.15 (0.19)	0.882	0.92	−0.05 (−0.25–0.15)
Irregular	1.26 (0.27)	0.958	1.25 (0.23)	0.927	0.91	−0.00 (−0.23–0.22)	1.26 (0.24)	0.875	0.94	0.01 (−0.18–0.20)
Obstacle	N/A	N/A	0.99 (0.25)	0.731	N/A	N/A	0.99 (0.24)	0.764	N/A	N/A
**Step time (ms)**										
Level	531 (44)	0.951	531 (45)	0.949	1.00	0.3 (−3.2–3.8)	537 (43)	0.873	0.96	6.3 (−18.3–31.0)
Irregular	540 (57)	0.957	540 (57)	0.952	1.00	0.6 (−2.8–4.0)	543 (55)	0.805	0.99	3.4 (−13.2–20.0)
Obstacle	733 (128)	0.934	715 (113)	0.804	0.98	−18 (−69–33)	703 (99)	0.700	0.86	−30 (−159–98)
**Cadence (steps/min)**										
Level	114 (9)	0.952	114 (10)	0.950	1.00	−0.0 (−0.9–0.8)	112 (9)	0.876	0.96	−1.4 (−6.7–4.0)
Irregular	112 (11)	0.953	112 (11)	0.948	1.00	−0.1 (−1.0–0.7)	112 (11)	0.774	0.99	−0.5 (−4.4–3.3)
Obstacle	84 (13)	0.919	86 (12)	0.718	0.98	1.9 (−3.8–7.6)	87 (12)	0.722	0.87	3.3 (−9.1–15.7)

*LOA, Limits of agreement; N/A, Not available. The crossing speed during the obstacle crossing condition could not be evaluated using video-derived data*.

### Validity

**Figure 3:**
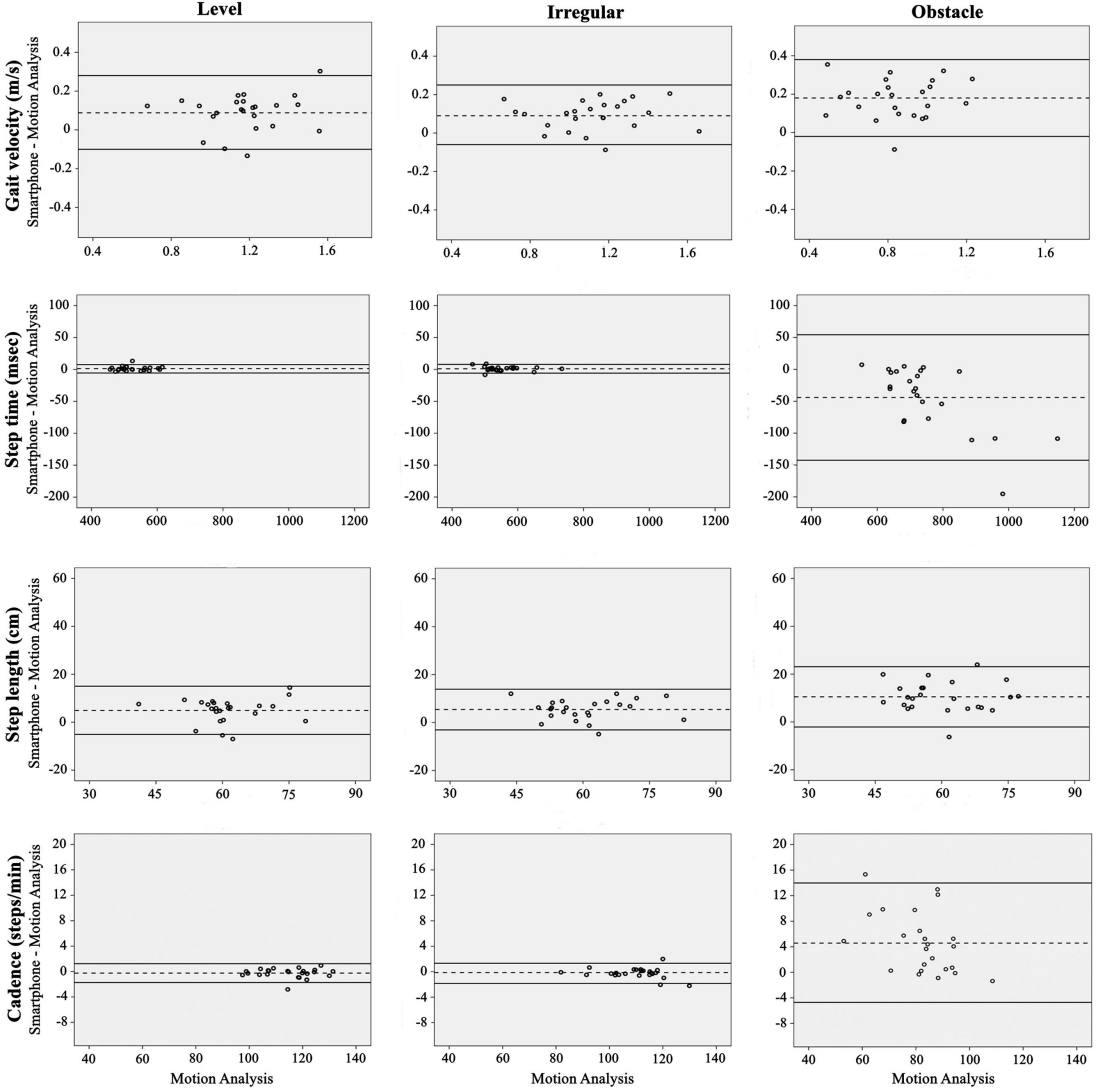
Bland Altman plots for gait velocity, step time, step length, and cadence when using the smartphone-based assessment compared to Motion Analysis in the laboratory environment. The dashed lines are the average difference, with the solid lines being the repeatability coefficient (±1.96 SD).

**Figure 4:**
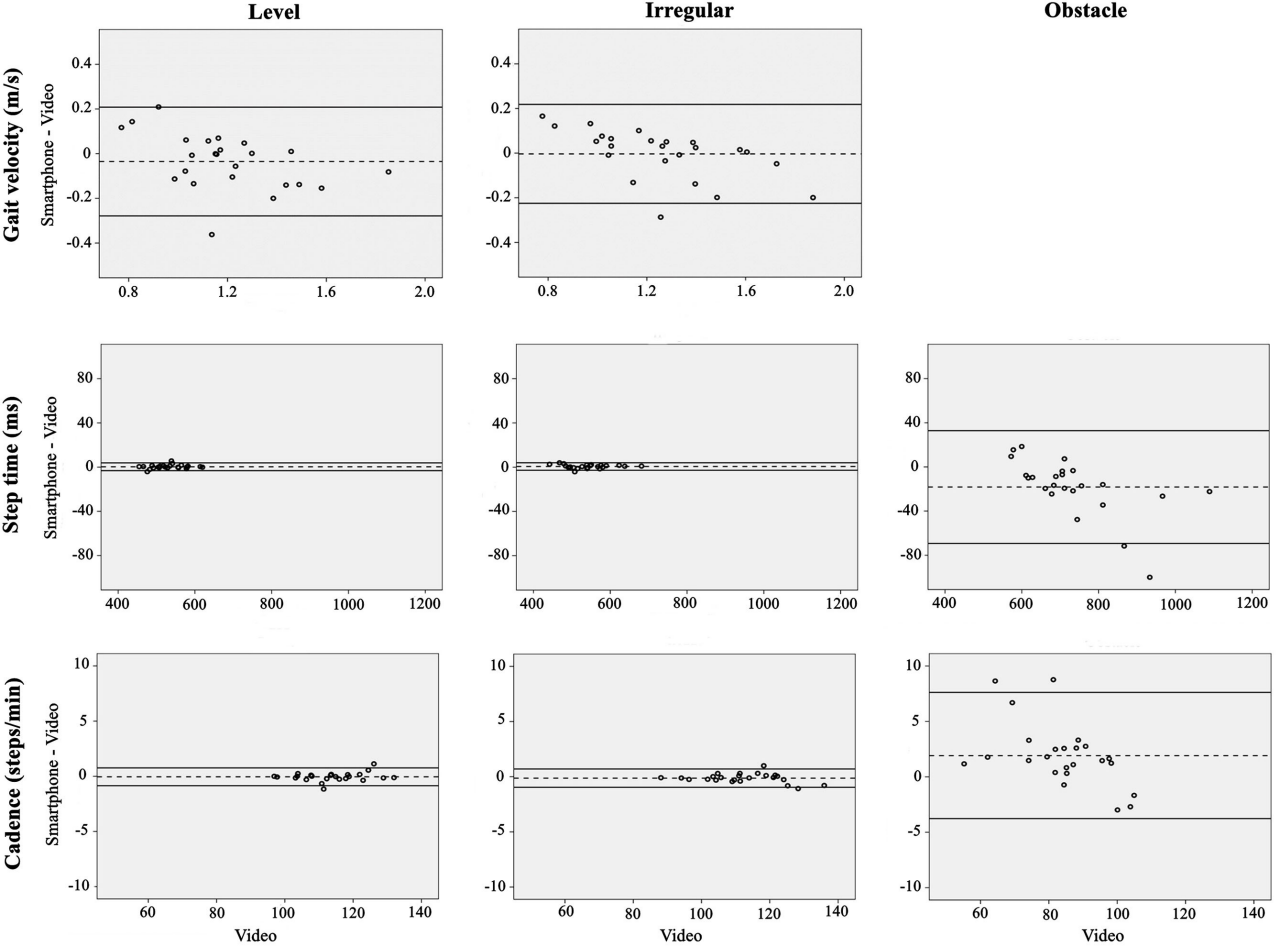
Bland Altman for gait velocity, step time, and cadence when using the smartphone-based assessment compared to video in the free-living environment. The dashed lines are the average difference, with the solid lines being the repeatability coefficient (±1.96 SD).

High to very high correlations were found for all spatiotemporal gait parameters when placing the smartphone on the body or bag, across all conditions and environments (*r* ranging from 0.81 to 1.00 and 0.72 to 1.00 for the body and bag, respectively; Tables 1, 2). Bland-Altman analysis revealed slightly increased bias and limits of agreement during the obstacle crossing condition, when compared to either the level or irregular surface walking for all parameters (Figures 3, 4; Tables 1, 2).

Low to high correlations (*r* ranging from 0.49 to 0.75) were demonstrated for the SI COM displacement, when the smartphone was placed on the body. Negligible to moderate correlations were found for the COM displacement along the AP and ML directions for the smartphone placed on the body. Furthermore, negligible to low correlations were found for COM parameters across all directions and conditions, when placing the smartphone in the bag.

### Effect of Age, Walking Condition, and Environment on Gait

Effects of age, condition, and environment were investigated using smartphones from both body and bag locations. However, as identical interaction and main effects were found for all gait parameters between the two phone locations, only data derived from the smartphone on the body location were reported (Table 3). Results revealed no significant three-way group × condition × environment interaction for gait velocity, step time, step length, or cadence. Furthermore, there was an absence of group × environment interactions as well as the main effect of group and environment for all parameters.

**Table 3:** Gait and balance parameters [mean (SD)] for young and older adults under three walking conditions in the laboratory and free-living environments.

**Condition**	**Young**		**Older**		***P*****-value**			
	**Laboratory**	**Free**	**Laboratory**	**Free**	**Gr × Cond × Env**	**Gr × Cond**	**Gr × Env**	**Cond × Env**
**Gait velocity (m/s)**								
Level	1.31 (0.20)	1.28 (0.28)	1.22 (0.28)	1.17 (0.18)	0.696	0.061	0.827	<0.001^*^
Irregular	1.31 (0.20)	1.42 (0.27)	1.11 (0.27)	1.22 (0.21)				
Obstacle	1.12 (0.19)	1.06 (0.21)	0.94 (0.24)	0.91 (0.27)				
**Step time (ms)**								
Level	523 (37)	524 (41)	533 (57)	538 (49)	0.488	0.005^*^	0.914	0.055
Irregular	534 (37)	527 (41)	573 (73)	554 (69)				
Obstacle	654 (57)	661 (57)	755 (116)	768 (132)				
**Step length (cm)**								
Level	68.3 (10.0)	66.3 (12.4)	64.1 (10.1)	62.3 (7.9)	0.822	0.182	0.609	0.002^*^
Irregular	69.8 (9.5)	73.9 (11.4)	62.2 (10.5)	66.8 (7.3)				
Obstacle	72.8 (11.9)	69.6 (11.9)	68.8 (10.0)	67.4 (14.2)				
**Cadence (steps/min)**								
Level	115 (8.2)	115 (9.1)	114 (11.8)	112 (10.1)	0.445	0.005^*^	0.733	0.054
Irregular	113 (7.8)	115 (8.9)	106 (12.1)	110 (13.4)				
Obstacle	93 (8.2)	91 (7.9)	81 (10.9)	81 (12.6)				
**COM SI (cm)**								
Level	3.1 (0.4)	3.9 (0.7)	2.9 (0.6)	3.6 (0.9)	0.921	0.057	0.682	0.006^*^
Irregular	3.4 (0.6)	4.9 (0.8)	3.1 (0.8)	4.4 (0.9)				
Obstacle	4.7 (0.8)	5.5 (1.0)	5.0 (1.2)	5.9 (1.4)				

*All values were derived from the smartphone placed on the body. COM, Center of mass; SI, Superior-inferior; Gr, Group; Cond, Condition; Env, Environment*;

* *P <0.05*.

Group × condition interactions were significant for step time and cadence. When comparing the age groups for each walking condition, older adults walked with a longer step time (*p* = 0.012) and lower cadence (*p* = 0.009) than young adults only during the obstacle crossing condition. No group differences were found during level walking or walking over an irregular surface. Condition × environment interactions were significant for gait velocity and step length. When comparing the environmental setting for each walking condition, there was an environmental effect only during the irregular surface walking condition, but not during the level walking and obstacle crossing conditions. For irregular surface walking, participants ambulated ~0.11 m/s faster (*p* = 0.001) and took ~4.3 cm longer steps (*p* = 0.004) in the free-living environment compared to the laboratory environment.

### Effect of Age, Walking Condition, and Environment on Balance

Due to poor validity, the AP and ML COM displacement was not analyzed for effects of age, condition, and environment. For the SI COM displacement, only the condition × environment interaction was found to be significant (Table 3). Participants walked with a larger COM displacement in the free-living environment (*p* < 0.001), compared to the laboratory, especially while walking over an irregular surface.

## Discussion

This study demonstrated that a smartphone can be used as a field-based assessment tool to quantify ambulatory tasks while navigating both unobstructed and obstructed surfaces. In support of our first hypothesis, smartphone-derived spatiotemporal gait parameters were valid and reliable while level walking, ambulating over an irregular surface, or crossing an obstacle, when placed on either the body or in a bag. Furthermore, high to very high validity and good to excellent reliability was demonstrated for spatiotemporal gait measures in both the laboratory and free-living environments. However, partially supporting our hypothesis that a smartphone-based assessment of the COM displacement would be valid and reliable across all three directions, results of our study revealed that only the vertical displacement of the COM could be quantified accurately when attached to the body.

The moderate to very high validity and excellent reliability results for gait parameters and the SI COM displacement found during level walking in this study were comparable to those previously reported (Furrer et al., 2015; Silsupadol et al., 2017, 2019). When compared to motion capture, it was shown that body and bag placement of a smartphone had high to very high validity (Pearson's r from 0.86 to 1.00 and from 0.87 to 1.00, for the body and bag, respectively) for all spatiotemporal measures in the current study. In comparison, a previous study reported r values from 0.82 to 1.00 and from 0.77 to 1.00, for body and bag placements, respectively (Silsupadol et al., 2019). Furthermore, the use of a smartphone to quantify gait parameters during level walking was shown to have excellent reliability when placed on the body and bag (ICC_2,1_ ≥ 0.95), with similar results demonstrated previously (ICC_2,1_ ≥ 0.90 and ≥ 0.79, for the body and bag, respectively) (Silsupadol et al., 2017).

During level walking, our study found moderate correlations (*r* = 0.63) and excellent reliability (ICC = 0.77) for the vertical COM displacement when the smartphone was placed on the body. In line with our results, Furrer and colleagues also showed similar findings (*r* ~0.63 and ICC ~0.74), when compared to motion capture (Furrer et al., 2015). Alternatively, validity and reliability of the COM displacement in the AP and ML directions were less successful. Even though no previous studies have investigated the validity of the AP and ML COM displacement during gait, similar trends for the COM displacement were demonstrated when utilizing an inertial measurement unit during ski skating. Compared to motion capture, COM displacement demonstrated root mean square errors of 2.4, 3.2, and 0.6 cm for the AP, ML and SI axes, respectively (Myklebust et al., 2015). This decreased accuracy for the AP and ML axes might be due to small misalignments of the accelerometer in relation to gravity affecting the transverse plane more than the vertical direction (Myklebust et al., 2015). For the bag location, the smartphone demonstrated poor validity and reliability for the COM displacement across all three axes. Along with possible misalignment and increased movement at this location, reduced validity may be due to the bag being placed over the right hip, rather than corresponding to the body's COM location (Kavanagh and Menz, 2008). In addition, while the L3 location has low transverse plane rotation, movement of the hip during walking might have caused extraneous movement of the bag, as movement of the bag was not restricted.

While previous work has mainly focused on unobstructed level walking, this is the first study to reveal very high validity and excellent reliability of using smartphones to measure spatiotemporal gait measures when ambulating over irregular walkways. The similar findings obtained from level and irregular surface walking indicate that the smartphone-based algorithm employed in this study is appropriate even for walking environments where gait is continuously challenged. Alternatively, slightly reduced validity and reliability was found during the obstacle crossing condition, particularly for the bag placement. This might be due to the small number of steps utilized for assessing obstacle crossing and the preference of leading limb. As the bag was placed over the right hip, choice of left or right limb to cross the obstacle might affect reliability and validity, since the hip flexion angle of the leading limb is greater than that of the trailing limb during obstacle crossing (Chou et al., 2001).

In partial support of our second hypothesis, smartphone-based assessment revealed age-related differences in gait and balance performance only during obstacle crossing. Older adults crossed the obstacle with a longer step time than young adults. Age-related differences may become more pronounced when a greater time constraint is imposed and a wider obstacle is utilized (Chen et al., 1994; Weerdesteyn et al., 2005). A smartphone-based gait assessment also revealed balance and gait changes depending upon the task and environment. During irregular surface walking, participants utilized a faster gait speed and a longer step length in the free-living environment, compared to the laboratory environment. Environmental differences in walking strategy while navigating the uneven surface may be due the perceived hazard of the terrain within each environment. Shorter steps taken while navigating the irregular surface in the laboratory might be due to the consistency of perturbation (~every 30 cm) and compliance of the plywood surface. Even though our irregular terrain in the laboratory was intended to be analogous to surfaces found outdoors, the irregular surface in the free-living environment was less uniform and cement. Unlike previous studies that found differences between laboratory and daily life gait when walking on a level surface (Brodie et al., 2016; Giannouli et al., 2016), this study found that the environment does not affect the way participants walk during this simple condition. With longer walking distances comprising activities of daily living, gait discrepancies between environments may be more evident.

There were a few limitations to the current study. While this study attempted to address a gap in the literature in regards to evaluating gait in the real-world environment, participants in this study were explicitly asked to walk a certain distance and were in the presence of research staff. Although gait trials utilized a 10-m walking distance, it is expected that longer duration walking bouts would lead to improved accuracy results, as misclassification and computation errors commonly occur during the acceleration and deceleration phases found at the beginning or end of trials, respectively (Lugade et al., 2014). Alternatively, this was the first study to explicitly evaluate gait and balance from smartphone-derived measures among young and older adults across various environments. Although utilizing accelerometer-based gait assessment under real-world conditions is beneficial, few studies have performed long duration evaluations in the free-living environment. Therefore, future studies need to investigate the feasibility of home-based smartphone evaluations of gait in an environment where participants freely engage in everyday walking. Additionally, investigation of additional participants with variable body sizes, ages, and morbidities would increase the robustness of this methodology.

In conclusion, given its ease-of-use, cost effectiveness, and portability, smartphones offer an alternative approach to analyze gait and subsequently provide an easy-to-implement tool for remote patient monitoring and home health care. In particular, smartphone-based assessment, regardless of phone placement, was shown to be reliable and valid for all spatiotemporal gait parameters across obstructed and unobstructed conditions, both indoors and outdoors.

## Data Availability Statement

The raw data supporting the conclusions of this article will be made available by the authors, without undue reservation.

## Ethics Statement

The studies involving human participants were reviewed and approved by Chiang Mai University Research Ethics Committee (number AMSEC-61EX-043). The patients/participants provided their written informed consent to participate in this study.

## Author Contributions

JK: conceptualization, methodology, validation, formal analysis, investigation, data curation, and wrote-original draft. PS: conceptualization, methodology, validation, formal analysis, investigation, wrote-reviewing and editing, supervision, and project administration. TK: methodology, validation, investigation, and data curation. VL: conceptualization, methodology, software, validation, formal analysis, investigation, writing-reviewing and editing, and project administration. All authors contributed to the article and approved the submitted version.

## Conflict of Interest

VL was employed by the company Control One LLC. The remaining authors declare that the research was conducted in the absence of any commercial or financial relationships that could be construed as a potential conflict of interest.
